# Testosterone increases apoptotic cell death and decreases mitophagy in Leber’s hereditary optic neuropathy cells

**DOI:** 10.1007/s13353-020-00550-y

**Published:** 2020-03-10

**Authors:** Elona Jankauskaitė, Anna Maria Ambroziak, Parvana Hajieva, Monika Ołdak, Katarzyna Tońska, Magdalena Korwin, Ewa Bartnik, Agata Kodroń

**Affiliations:** 1 Institute of Genetics and Biotechnology, Faculty of Biology, University of Warsaw, 5a Pawińskiego Str., 02-106 Warsaw, Poland; 2 Faculty of Physics, University of Warsaw, 5 Pasteur Str., 02-093 Warsaw, Poland; 3 Institute for Pathobiochemistry, University Medical Center of the Johannes Gutenberg University Mainz, Duesbergweg 6, D-55099 Mainz, Germany; 4 Department of Genetics, Institute of Physiology and Pathology of Hearing, 10 Mochnackiego Str., 02-042 Warsaw, Poland; 5 Department of Histology and Embryology, Center of Biostructure Research, Medical University of Warsaw, 5 Chałubińskiego Str., 02-004 Warsaw, Poland; 6 Department of Ophthalmology, Medical University of Warsaw, 13 Sierakowskiego Str., 03-709 Warsaw, Poland

**Keywords:** LHON, Apoptosis, Mitophagy, Testosterone, Cell death

## Abstract

Leber’s hereditary optic neuropathy (LHON) is one of the most common mitochondrial diseases caused by point mutations in mitochondrial DNA (mtDNA). The majority of diagnosed LHON cases are caused by a point mutation at position 11,778 in the mitochondrial genome. LHON mainly affects young men in their 20s and 30s with usually poor visual prognosis. It remains unexplained why men are more likely to develop the disease and why only retinal ganglion cells are affected. In this study, a cell model was used for the first time to investigate the influence of testosterone on the cell death mechanism apoptosis and on an autophagy/mitophagy. Cells with m.11778G > A were found to be significantly more susceptible to nucleosome formation and effector caspase activation that serve as hallmarks of apoptotic cell death. Cells having this mutation expressed higher levels of mitophagic receptors BNIP3 and BNIP3L/Nix in a medium with testosterone. Moreover, cells having the mutation exhibited greater mitochondrial mass, which suggests these cells have a decreased cell survival. The observed decrease in cell survival was supported by the observed increase in apoptotic cell death. Autophagy was analyzed after inhibition with Bafilomycin A1 (Baf A1). The results indicate impairment in autophagy in LHON cells due to lower autophagic flux supported by observed lower levels of autophagosome marker LC3-II. The observed impaired lower autophagic flux in mutant cells correlated with increased levels of BNIP3 and BNIP3L/Nix in mutant cells.

## Introduction

Leber’s hereditary optic neuropathy (LHON) is a maternally inherited form of an incurable bilateral painless vision loss due to isolated atrophy of the optic nerve caused by point mutations in mitochondrial DNA (mtDNA, Man et al. 2003). LHON occurs mostly in young adults affecting both eyes simultaneously or sequentially over a period of a few months or weeks with usually very poor visual prognosis (Man et al. 2003; Puomila et al. 2007; Gueven et al. 2017; Devi et al. 2017). One of the unexplained traits of the disease is the fact that men are 4–5 times more likely to develop the disease than women (Kirkman et al. 2009). The three most common mtDNA point mutations (m.11778G > A, m.3460G > A and m.14484 T > C) account for over 90% of diagnosed LHON cases (Puomila et al. 2007). In most cases, mtDNA is 100% mutated in every cell, but only retinal ganglion cells (RGCs) are affected. The cells from affected material are unavailable for experiments; therefore, cell models are used to study LHON. The most common models are lymphoblasts, fibroblasts, and cybrids (Jankauskaitė et al. 2017). Peripheral blood mononuclear cells (PBMC) are used to establish lymphoblast cell lines that have mainly been used for biochemical analyses, viability assays, or to compare the degree of OXPHOS (oxidative phosphorylation) impairment in LHON. PBMCs, similarly to RGCs, have a metabolism mainly sustained by OXPHOS and therefore represent a good model to be investigated in LHON research (Falabella et al. 2016).

Mitochondria are essential in the initiation of apoptosis, as they release cytochrome c into the cytosol during the first stage of apoptosis, activating caspase 9 and a protein called bcl-2, located in the outer mitochondrial membrane. Subsequently, the inner mitochondrial membrane potential decreases, which is mediated by the opening of the mitochondrial permeability transition pore (mPTP). It is worth pointing out that autophagy is a process to maintain cellular homeostasis (by removing damaged parts of the cell or organelles); therefore, autophagy is not considered as a cell death mechanism itself. Over-stimulation of autophagy may act as another cell death mechanism, which has recently been widely studied; it mediates the degradation and recycling of intracellular components, including damaged organelles or protein aggregates, to sustain cell homeostasis (Boya et al. 2013). Autophagy is also responsible for the removal of whole damaged mitochondria in a process called mitophagy. This pathway can also mediate degradation of mitochondria in developmental contexts, during a process called programmed mitophagy (Ney 2015).

Hormonal differences are one of the main causes proposed to the explain gender bias in LHON (Giordano et al. 2011; Pisano et al. 2015). Oestradiol is mainly produced in the ovary and white adipose tissue in women (Nelson and Bulun 2001). Testosterone is produced mainly in Leydig cells of testes in males resulting in very high local concentrations, and in ovaries in females – this testosterone is later released to the bloodstream (Handelsman et al. 2018). The brain is also believed to be a steroidogenic organ that expresses molecules and enzymes necessary for the conversion of cholesterol into sex hormones such as progesterone, oestradiol, and testosterone. In the retina, cholesterol was found to be transformed into pregnenolone and then to sex steroids: estrogen or testosterone. Subsequently, it was discovered that estradiol is formed locally in the retina via cholesterol-based synthesis and testosterone aromatization (Cascio et al. 2015, 2007). Moreover, high concentrations of testosterone (1–10 μM) were found to initiate an apoptotic pathway and induce neurotoxicity in neuroblastoma cells (Estrada et al. 2006). Testosterone and 5-α-dihydrotestosterone (5α-DHT) bind with high affinity to androgen receptors (AR). The ligand-AR complex modulates the expression of several nuclear and mitochondrial target genes, which together, promote fatty acid oxidation, oxidative phosphorylation, and mitochondrial biogenesis, attenuating the generation or accumulation of ROS (reactive oxygen species) (Pitteloud et al. 2005). Testosterone levels in men are at their highest during adolescence and early adulthood and decline about 1% per year after age 30 (Stanworth and Jones 2008). This fact fits the age of typical LHON disease onset. Moreover, it was demonstrated that 17-β oestradiol and testosterone have opposite effects on mitochondrial biogenesis in white adipocyte cells (Capllonch-Amer et al. 2013).

Mitochondria play an essential role during the initial steps of sex steroid hormone biosynthesis. Receptors for estrogen and androgen hormones have been detected in mitochondria of various cell types (Psarra and Sekeris 2008). The role of these receptors in mitochondrial transcription, OXPHOS, biosynthesis, and apoptosis is not yet clear. The effects of estrogens on various cell processes have been described, but information about testosterone effects is very limited. Therefore, the main goal of this study was to analyze the effects of testosterone in apoptosis and autophagy/mitophagy. Lymphoblast cell lines were established from affected individuals and controls to maintain nuclear genomes and mitochondrial genomes from “original” cells. Cells were initially treated with 3 different concentrations of testosterone varying from 10 nm to 1 μM as the concentration in adult men varies from 7 to 30 nm/L. However, in subsequent experiments, it was decided to use only 10 and 100 nm concentrations as additional testosterone did not seem to increase the effects on observed parameters in lymphoblasts.

## Materials and methods

### Patients and controls

The samples used in this study were immortalized lymphoblasts established from peripheral blood samples. Blood samples were obtained in collaboration with SPKSO Ophthalmic University Hospital in Warsaw after obtaining informed consent. Control cell lines were established from age-matched healthy volunteers (20 to 30 years old) from the Institute of Genetics and Biotechnology, Faculty of Biology, University of Warsaw. In this study, we used 4 cell lines from affected m.11778G > A men, 3 cell lines from control men, and 3 cell lines from 3 control women. We did not have access to affected m.11778G > A women material to establish cell lines; however, we had 2 cell lines from unaffected m.11778G > A women carriers. All LHON cases were diagnosed by an ophthalmologist and were confirmed to be carriers of the m.11778G > A mutation. The project received permission KB/187/2015 from the Bioethics Committee of Warsaw Medical University.

### B lymphocyte isolation and immortalization

Peripheral blood samples were collected in BD Medical Vacutainer™ Glass Blood Collection tubes (Becton Dickinson, cat. #364606, United States). Within 24 h after blood collection, 3-5 mL of blood was transferred to Leucosep™ separation tube (Greiner Bio-One GmbH, cat. #136288, Germany) and centrifuged for 15 min at 25 °C at 800 g. Next, the transparent layer enriched in B lymphocytes was carefully collected and transferred to a new Falcon tube, followed by two washing cycles in 10 mL of PBS at 25 °C at 250 g. The immortalization procedure requires transformation of cells using Epstein-Barr virus (EBV, cotton-top tamarin strain B95–8, Sigma Aldrich, cat.#85011419-1VL, Poland). After centrifugation, sediment (B lymphocyte fraction) was dissolved in 4 mL of cell culture medium and 1 mL of previously prepared EBV supernatant. Cyclosporine-A (Sigma Aldrich, cat.#30024, Poland) dilution 1:50 was included to inhibit the T lymphocytes. After the procedure, cells were transferred to 25cm^3^ cell culture flask (VWR™, cat.# 10062-870, Germany) for 8 days. After 8 days, fresh cyclosporine-A solution was added, and cells were kept for another 7 days. After 7 days, half of the cell culture medium was changed. Two weeks after the immortalization if the cells started to attach to each other forming small clumps the procedure was considered to be successful.

### Cell count and viability assay

Cells were cultured in RPMI-1640 medium (Thermo Fisher Scientific™, cat. #11875093, United States) supplemented with 1 mM Gibco™ Sodium Pyruvate (Thermo Fisher Scientific™, cat. #11360070, United States), 1% HEPES (Thermo Fisher Scientific™, cat. #15630080, United States), 10% Gibco™ FBS (Thermo Fisher Scientific™, cat. #10500064), 1% penicillin/streptomycin (Thermo Fisher Scientific™, cat. 15140122, United States), and 0.05 mg/mL uridine (Sigma-Aldrich™, cat. #U3003, Germany). Cells were kept at 37 °C in a humidified incubator with 5% CO2. Viability and density of the cells was counted using the Trypan Blue dye exclusion assay (Thermo Fisher Scientific™, cat. #1520061, United States). Cell lines used in the study were checked for contamination with mycoplasma using EZ-PCR™ Mycoplasma Test Kit (BI Biological industries, cat. 20-700-20, United States).

### Formation of nucleosomes

Apoptosis was analyzed using Cell Death Kit Elisa Plus (Roche™ cat. #11544675001, Germany), following the manufacturer’s instructions. For every analysis, 1 × 10^4^ cells were used, every experiment was repeated three times. Absorbance was measured at 405 nm in a Paradigm Detection Platform (Beckman Coulter) plate reader. Enrichment of nucleosomes in the cytoplasm of the cells treated with different concentrations of testosterone was analyzed using two different cell culture media – a standard one (complete RPMI-1640 medium) and medium without glucose (RPMI-1640 no glucose, Thermo Fisher Scientific™, cat. #11879020, United States) supplemented with 5 mM galactose to force cells to rely more on ATP generated via OXPHOS. Experiments performed on a cell model and lymphoblasts are not the target cells for testosterone, therefore we decided to use 3 different concentrations of testosterone in our experiments – hypogonadal 10 nM and a higher concentration of 100 nM as it is very likely some effects due to limits of our cell model would not be possible to detect. Cells were kept 4 and 24 h in the medium at 37 °C before analysis.

### Activation of caspases 3 and 7

To support findings of apoptosis, Caspase 3 and 7 activation was measured using commercially available Caspase-Glo™ 3/7 Assay (Promega cat. #G8090, United States) following the manufacturer’s instructions. For every analysis, we used 1 × 10^4^ cells, every experiment was repeated three times. Conditions used for the analysis were the same as described previously. Luminescence was measured using a Paradigm Detection Platform (Beckman Coulter) plate reader.

### Mitochondrial mass

Mitochondrial mass was measured using Mitotracker Green FM probes (Thermo Fisher Scientific™, cat. #M7514, United States). Cells were plated on 24-well plates at a density of 1 × 10^5^ cells. Cells were incubated with pre-warmed medium containing 200 nM Mitotracker dye at room temperature for 20 min, followed by two washing steps. Fluorescence was measured at 485–535 nm in a Paradigm Detection Platform (Beckman Coulter) plate reader. The fluorescence of the samples was calculated against cells not stained with the dye (autofluorescence).

### Immunoblotting

Proteins were extracted from the samples using Pierce™ RIPA lysis buffer (Thermo Scientific™, cat. #389900, United States) enriched with a cocktail of protease and phosphatase inhibitors (Thermo Scientific™, cat. #88669, United States) followed by mechanical extraction keeping lysates on ice at all times during the isolation procedure. Protein concentration was measured using the Bradford Protein Assay Kit (BIO-RAD™, cat. #5000001, United States), according to the manufacturer’s instructions. Twenty to thirty micrograms of protein lysates from each sample were loaded onto 10–15% PAGE mini gels depending on the molecular weight of the protein of interest. Proteins were transferred to Amersham™ Protran™ 0.22 μM nitrocellulose membrane (GE Healthcare Life Science, cat. #10600002, Germany). Next, membranes were blocked in 5% non-fat dry milk in PBS for 1 h at room temperature, washed 5 min with PBS-Tween (PBST) containing 1% Tween™ 20 Surfact-Amps™ Detergent Solution (Thermo Scientific™, cat. #85113, United States) three times. Membranes were incubated with specific primary antibodies (dilution 1: 1000 for all antibodies used) – anti-Bnip3L/Nix (Cell Signaling Technology, INC., cat. #12396), anti-Bnip3 (Cell Signaling Technology, INC., cat. #44060) for mitophagy receptors, anti-LC3 (Cell Signaling Technology, INC., cat. #12741) and p62 (Cell Signaling Technology, INC., cat. #95697) for autophagy, anti-COX IV (Cell Signaling Technology, INC., cat. #4844) served as a mitochondrial loading control, and anti-β actin (Cell Signaling Technology, INC., cat. #8457) served as an internal loading control. Overnight incubation with primary antibodies was followed by three washing steps with PBST for 5 min at room temperature. Later, blots were incubated with secondary, polyclonal anti-rabbit (Sigma™, cat. #SLBP3451V, United States) antibodies (dilution 1: 10,000) for 1 h at room temperature followed by three washing steps. Blots were visualized with Clarity™ Western ECL Substrate solution (BIO-RAD™, cat. #190–5061, United States) in FluorChem Q quantitative western blot imaging from protein sample system (ProteinSimple™). For proteins with molecular weight (MW) less than 20 kDa, a three-layer tricine PAGE system was used. The level of detected proteins was quantified using ImageJ software, normalized on the basis of cell-equivalents.

### Statistical analysis

Western blot pictures were analyzed using ImageJ software. Protein immunoreactivity within each blot was normalized to untreated control sample, then the data was combined from 3 independent different blots. Data obtained from measured absorbance, luminescence, and fluorescence were also normalized to untreated control sample for each experiment, then the data from all 3 independent experiments were combined. Differences in groups were compared according to cell line sex. For data calculated within men/women groups, student *t* test was used (two-tailed). *P*-values were rounded to three decimal places. *P*-values less than 0.001 were reported as < 0.001. Obtained differences were considered to be significant when the *p* value was * < 0.05, ** < 0.01, and *** < 0.001.

## Results

**Fig. 1:**
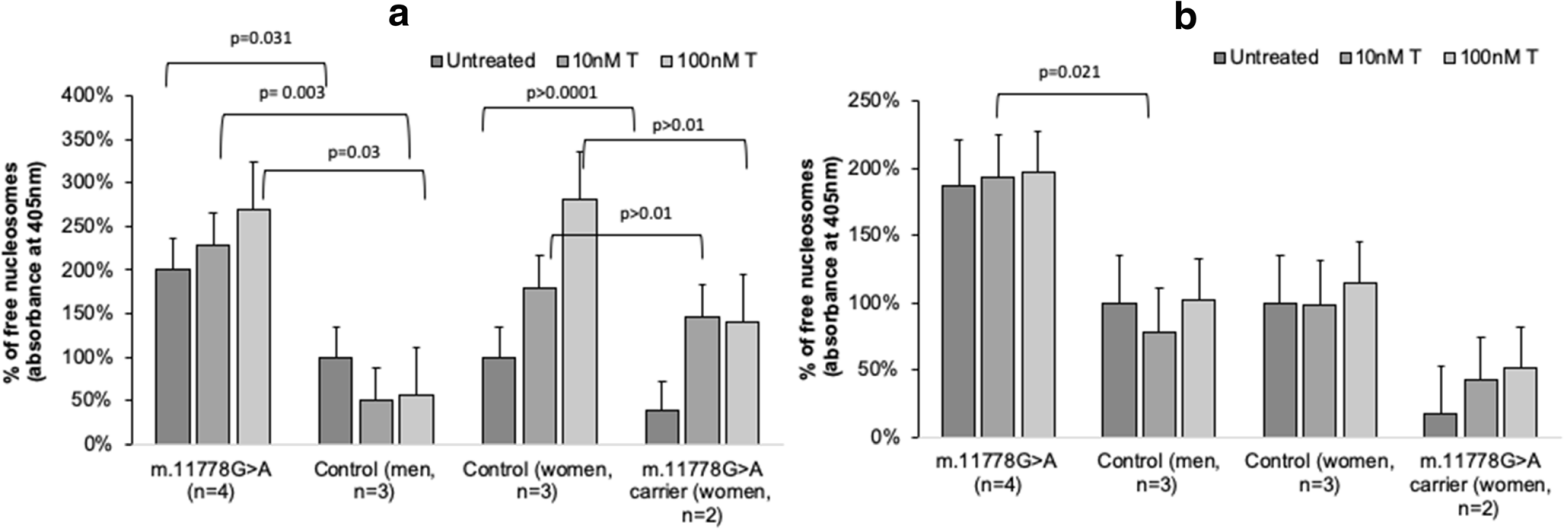
Effect of testosterone on formation of cytoplasmic DNA-histone nucleosome complexes. Cells were incubated with 10 nM and 100 nM concentrations of testosterone (T), 4 different cell line groups were used – m.11778G > A lymphoblasts from affected individuals (XY), Controls (XY, XX), and m.11778G > A unaffected carriers (XX). **a**. Nucleosome formation in cells grown in complete medium for 4 h. **b** Nucleosome formation in cells grown in medium without glucose supplemented with 5 mM galactose. Measured absorbance (405 nm) was normalized to untreated control sample according to cell line sex (affected m.11778G > A (XY)/Control (XY), m.11778G > A carriers (XX)/Control (XX)). Data represented as a mean value ± SD where each experiment was repeated 3 times for each cell line analyzed. For data compared within men/women groups multifactorial ANOVA *p* values are shown on the graph

We investigated the relationship between free nucleosome formation and effector caspase activation in m.11778G > A and control cells cultured with and without testosterone. In particular, we examined whether LHON cells were more likely to undergo apoptosis after treatment with concentrations of testosterone varying from physiological to supraphysiological levels (Fig. 1).

We observed that lymphoblasts with the m.11778G > A mutation from affected men were approximately 6 times more likely to undergo apoptosis than cells from control men after 4 h in complete medium with an almost two-fold increase in the remaining conditions (Figs. 1a, b). At the same time, we observed reduced levels of apoptotic cells in women m.11778G > A mutation carriers compared to control women (Figs. 1a, b). Moreover, increasing levels of apoptosis in our examined conditions also correlated with increasing concentration of testosterone.

**Fig. 2:**
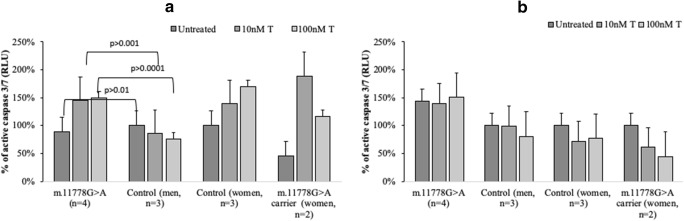
Effect of testosterone on activation of effector caspase 3 and 7. Cells were incubated with 10 nM and100nM concentrations of testosterone (T), 4 different cell line groups used – m.11778G > A lymphoblasts from affected individuals (XY), Controls (XY, XX), and m.11778G > A unaffected carriers (XX). **a**. Caspase 3/7 activation in cells grown in complete medium for 4 h. **b**. Caspase 3/7 activation in cells grown in medium without glucose supplemented with 5 mM galactose. Luminescence was normalized to untreated control sample according to cell line sex (affected m.11778G > A (XY)/Control (XY), m.11778G > A carriers (XX)/Control (XX)). Data represented as a mean value ± SD where each experiment was repeated 3 times. For data compared within men/women groups multifactorial ANOVA *p* values are shown on the graph

Apoptosis, an efficient cell death program, is mediated through the intrinsic or extrinsic pathway as a response to apoptosome stimuli. Both pathways initially lead to the activation of caspases. We observed that m.11778G > A lymphoblasts cultured in complete medium or in medium with 5 mM galactose, exhibited increased activity of effector caspases 3 and 7 (Figs. 2a, b). Unaffected women m.11778G > A carriers exhibited almost two-fold lower activation of caspases when not treated with testosterone (Fig. 2a), this observation supports the observed reduced levels of apoptosis in these cells.

**Fig. 3:**
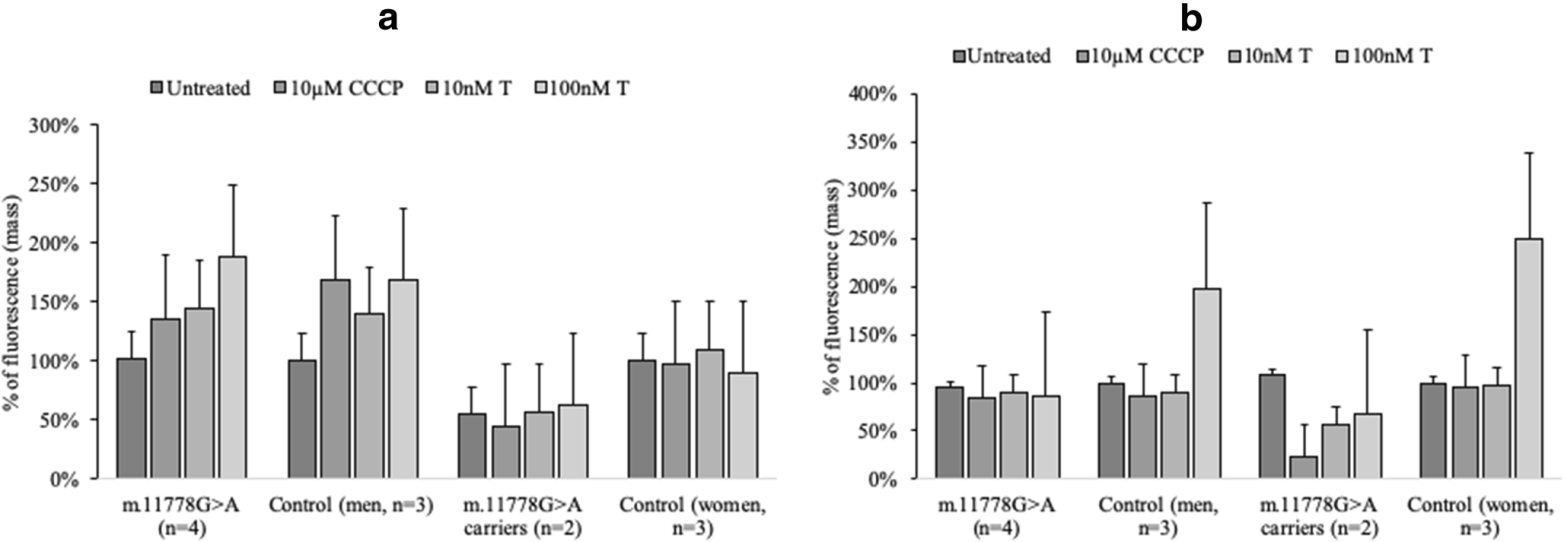
Effect of testosterone on mitochondrial mass in m.11778G > A and control lymphoblasts. Cells were incubated with 10 nM and 100 nM concentrations of testosterone (T), autophagy was induced by 10 μM CCCP. Fluorescence was measured at 485-535 nm. **a.** Cells grown in complete medium for 4 h. **b**. Cells grown in complete medium for 24 h. Fluorescence was normalized to untreated control sample according to cell line sex (affected m.11778G > A (XY)/Control (XY), m.11778G > A carriers (XX)/Control (XX)). Data represented as a mean value ± SD where each experiment was repeated 3 times. For data compared within men/women groups multifactorial ANOVA was used

Cells with the m.11778G > A mutation from affected individuals have a higher apoptosis rate as measured by nucleosome formation. Petrovas et al. (2007) suggested that mitochondria may act as an amplification step for apoptosis. Therefore we investigated changes in mitochondrial mass. Increased mitochondrial mass is believed to be characteristic for cells with mitochondrial dysfunction (Márquez-Jurado et al. 2018; Redmann et al. 2017). No significant change was observed in mitochondrial mass after 4 h of incubation; however, LHON cells were shown to have a tendency to have higher mitochondrial mass (Fig. 3a). This effect was difficult to see since the mitochondrial mass in apoptotic cells is already high. However, women m.11778G > A mutation carriers had significantly reduced mitochondrial mass compared to control women (Figs. 3a, b).

**Fig. 4:**
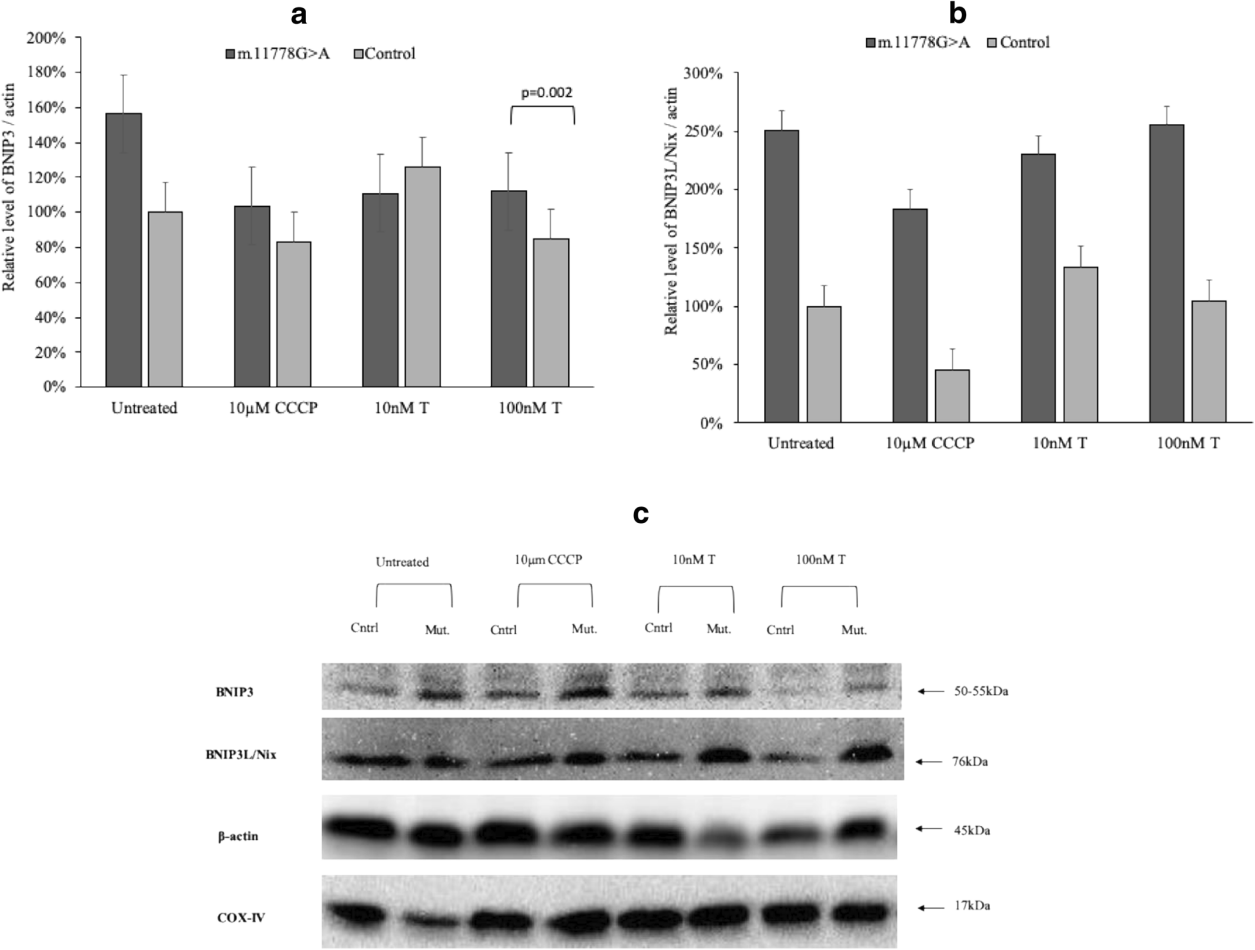
Effect of testosterone on mitophagy receptors. Cells were incubated with 10 nM and 100 nM testosterone (T) in standard medium for 4 h, for autophagy induction 10 μM CCCP was used. **a**. Relative level of BNIP3 protein. **b**. Relative level of BNIP3L/Nix protein. **c**. Western-blot analysis for BNIP3L/Nix and BNIP3, mitochondrial proteins COX IV, β actin served as loading control – in m.11778G > A and control lymphoblasts. Protein immunoreactivity within each blot was normalized to untreated control sample, then the data was combined from 3 independent different blots. Data represented as the mean value ± SD where each experiment was repeated 3 times. For data shown in panels student *t* test was used: ***p < 0.0001

In a female mouse model, the female brain has an innate ability to upregulate mitophagy, which protects neurons from death. Induction of autophagy/mitophagy may be another component of sex-specific compensatory mechanisms to prevent accumulation of oxidatively damaged cellular components, protecting against mitochondrial dysfunction (Demarest et al. 2016). In this study, for the first time we assessed the levels of two mitophagy receptors – BNIP3L/Nix and BNIP3. In lymphoblasts with the m.11778G > A mutation from affected men, levels of BNIP3L receptor tend to be almost two times higher than in men controls (Fig. 4b). However, it seems that testosterone might reduce mitophagy in cells with the LHON mutations. The observed increase in apoptotic cell death suggests that these cells try to compensate the mitochondrial dysfunction and promote the turnover of damaged mitochondria via mitophagy. Unfortunately, the presence of testosterone decreases the observed levels of BNIP3 proteins suggesting these cells are affected by testosterone and the mitochondrial network collapses (Fig. 4a).

It is worth noting that BNIP3L/Nix and BNIP3 are bifunctional mitochondrial proteins: they contain a BH3 domain with homology to the BH3-only pro-apoptotic members of the Bcl-2 family proteins and thus can induce cell death following mitochondrial membrane permeabilization (Esteban-Martínez et al. 2017; Ma and Liu 2018). The cell model used in this study should help mimic the processes taking place in RGCs, and, recently, it was reported that during retinal development, tissue hypoxia triggers a process resulting in increased expression of the mitophagy receptor BNIP3L/NIX (Esteban-Martínez et al. 2017). Moreover, there are some differences in initiation of autophagy between male and female cells. Du et al. (2009) demonstrated that female neurons can survive longer under starvation conditions before entering autophagy than male neurons.

**Fig. 5:**
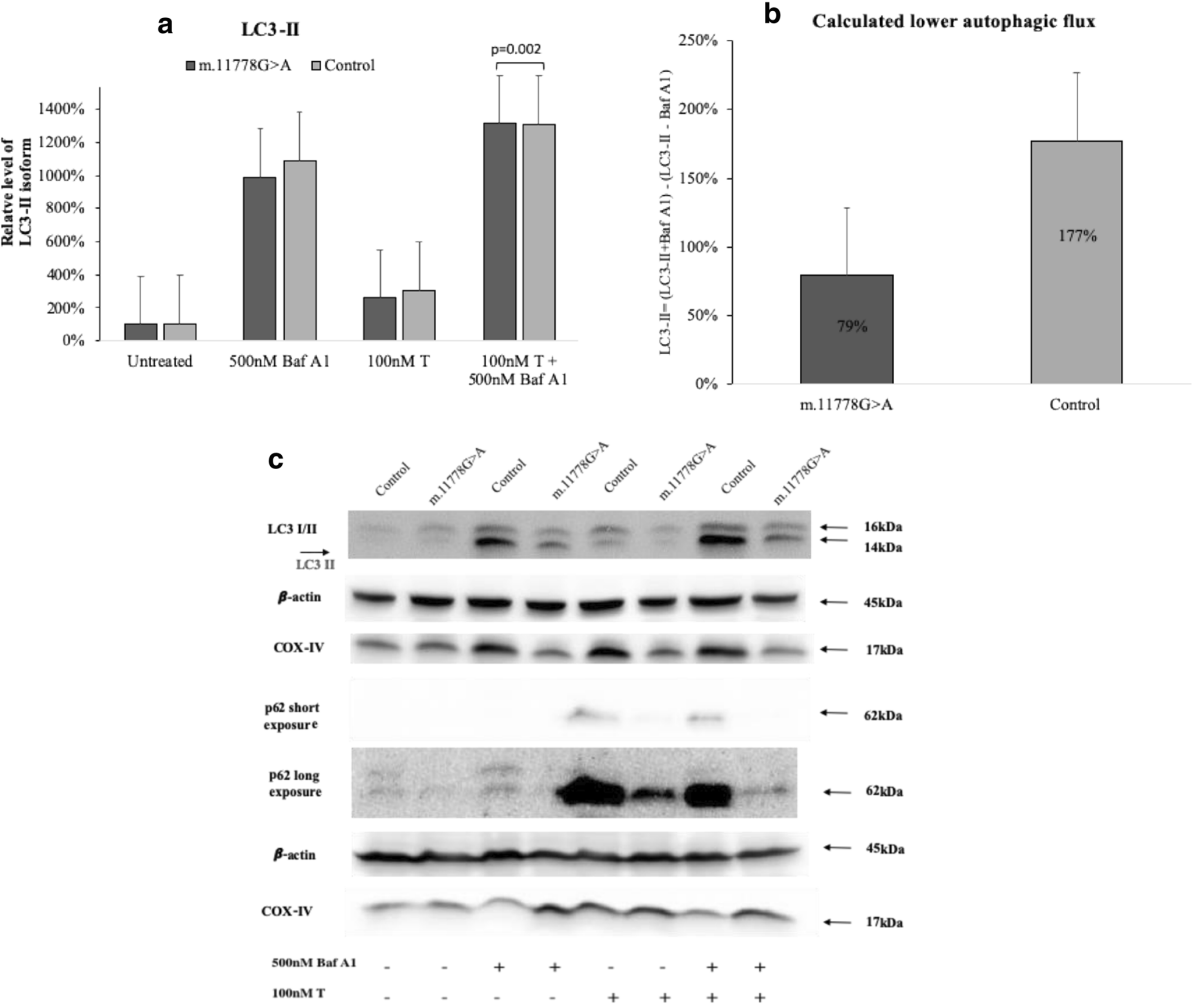
Effect of testosterone and Bafilomycin A1 on mitophagy receptor autophagy in m.11778G > A and control lymphoblasts. Cells were grown in standard medium for 4 h with 100 nM testosterone (T), for autophagy inhibition 500 nM Bafilomycin A1 used. **a**. Relative level of LC3 I and II isoform. **b**. Lower autophagy flux in m.11778G > A and control lymphoblasts. **c**. Western-blot analysis for p62, COX IV, β actin served as loading control. Protein immunoreactivity within each blot was normalized to untreated control sample, then the data was combined from 3 independent different blots. Data represented as the mean value ± SD where each experiment was repeated 3 times. For data shown in panels student *t* test was used: **p < 0.001

The main marker used to assess autophagy in the cells is LC3 protein, which exists in two isoforms within the cells – LC3-I and LC3-II, where the latter is often used as a biomarker for autophagy. However, this protein should be degraded at a later stages of autophagy, therefore, its accumulation may indicate inhibition of autophagy flux. This makes difficult to analyze the LC3-II levels. To further analyze the effects of testosterone on autophagy, a set of experiments using Bafilomycin A1 (Baf A1, Sigma, cat. #88899-55-2) was performed using m.11778G > A lymphoblasts from men and from control men. Baf A1 is used to impair fusion between autophagosomes and lysosomes (inhibit autophagy) and results in accumulation of autophagosomes in cells. Accumulation of LC3-II isoform serves as the marker of autophagy inhibition after Baf A1 disrupts the fusion of autophagosomes with lysosomes. To confirm lysosomal degradation of autophagosomes, the p62 marker was used as. Lysosomal degradation of autophagosomes leads to a decrease in the observed levels of p62. After trying several different concentrations, we decided to use 500 nM Baf A1 that was sufficient to inhibit autophagy in lymphoblast cells (Figs. 5a, c).

Autophagy inhibition was shown to decrease mitochondrial bioenergetics in intact neurons (Redmann et al. 2017), and it is linked to decreased mitochondrial Complex I, II, and IV substrate linked respiration. Decreased autophagy in dysfunctional mitochondria might promote further mitochondrial damage that could lead to degeneration of RGCs. In our study, we observed a trend of lower levels of LC3-II isoform accumulation after Baf A1 treatment in LHON cells compared to controls (Fig. 5c) that could indicate a lower level of autophagosome formation in the cell; however, this change was not considerable, and this was only preliminary observation that requires further investigation. The same trend was observed for p62 levels – after inhibition of autophagy we observed higher levels of p62 in control cells than in cells with the m.11778G > A mutation. This supports the observations of LC3-II levels. In addition, we found that after treatment with Baf A1 and 100 nM testosterone, we still observed lower autophagy induction in patient cells (Figs. 5b, c). Lower autophagic flux was measured in mutant cells (Fig. 5b), and we found a trend of an increased level BNIP3L/Nix (*p* = 0.001) in mutant cells as a result of an insufficient autophagic flux was observed.

## Discussion

We describe here for the first time the effects of testosterone on cell death pathways in LHON cells. Our results confirmed the findings reported previously – LHON m.11778G > A cells were more prone to undergo apoptosis than control cells without the m.11778G > A mutation. Also, we demonstrated that after treatment with testosterone within the physiological and supraphysiological range, cells are more prone to form nucleosomes and initiate apoptosis. The second important finding of this study was that activation of effector caspases 3 and 7 is also much more likely to happen in cells of affected individuals with the m.11778G > A mutation. Our result is not in agreement with data previously published by other researchers (Ghelli et al. 2003; Zanna et al. 2005), but we have used a different method to assess the activation of caspases and a different cell model, which maintained the nuclear genomes of individuals. Moreover, we found that the sex of the cell could impact the data. Our observations and the disparity of the results compared to the cybrid cell model, imply there are other compensatory mechanisms in women, and that the female nucleus of rho0 cells, used to obtain cybrids, masks some effects of the m.11778G > A mutation. Caspase inhibitors are reported to inhibit apoptosis in many different cell types, but it was demonstrated in vitro that apoptosis in retinal cell cultures happened via a caspase-independent pathway. An apparent discrepancy between our data and the observations reported by other authors (Ghelli et al. 2003; Zanna et al. 2005) concerns the activation of caspases during apoptotic cell death. In another study, it has been shown that caspases are oxidatively inactivated during retinal cell death, when apoptosis was induced via ROS mediators (Carmody and Cotter 2000). Almost two decades ago, it was documented that LHON cybrid cell death is apoptotic and is initiated via cytochrome c release, demonstrating the involvement of mitochondria in initiation of apoptosis. Also, LHON cybrids were shown to have increased ROS production (Ghelli et al. 2003; Zanna et al. 2005; Wong et al. 2002). These findings suggested that RGC degeneration could be caused by the higher ROS production levels in the mitochondria of the optic nerve. In addition, analyses of cells from LHON patients have shown that after induction of oxidative stress, apoptotic cell percentage increases almost two-fold compared to control cells (Battisti et al. 2004; Carmody and Cotter 2000), suggesting that LHON cells are more prone to undergo apoptosis.

Moreover, we found that cells with the LHON mutation also tend to have more mitophagic receptors, which play an important role in the normal function of RGC cells. Cells with this mutation were also found to have greater mitochondrial mass. It is worth noting that in many cases a statistically significant change was observed after 4 h, but not after 24 h, which might partially be explained by increased cell death which might affect observations after 24 h. A significant induction of the LC3-II isoform was observed after treatment with Baf A1 in controls, but not in m.11778G > A lymphoblasts after treatment with testosterone. Moreover, lower levels of the autophagosome marker LC3-II in LHON cells after autophagy inhibition suggested impairment of autophagy in the cells with the m.11778G > A mutation. The observed decrease in p62 levels after autophagy inhibition by BafA1 in conditions with additional testosterone treatment suggests these cells might have reduced capacity to remove damaged mitochondria via autophagy. The observed impaired lower autophagic flux in mutant cells correlated with the increased levels BNIP3L/Nix in mutant cells. This finding implies that impaired autophagy in cells with a mutation in mtDNA could cause accumulation of dysfunctional mitochondria that later leads to increased cell death that was also observed in this study.
